# Chronic laryngopharyngeal vagal neuropathy^☆^

**DOI:** 10.1016/j.bjorl.2018.04.001

**Published:** 2018-04-24

**Authors:** Michael S. Benninger, Andrea Campagnolo

**Affiliations:** a Chairman, Head and Neck Institute, The Cleveland Clinic, Cleveland, United States; b Self-employed Researcher, Rio de Janeiro, RJ, Brazil

Dysphonia, vocal fold paresis, laryngopharyngeal reflux disease (LPRD), chronic cough, globus, and laryngospasm are all common disorders seen by otolaryngologists. There is clear overlap in these disorders in some patients but in other patients the relationships are not so distinct. Is reflux a primary mediator of chronic cough, globus and laryngospasm or is there something far more fundamental accounting for these associations? Are these distinct overlapping disorders or is there a common association? The term “Chronic laryngopharyngeal neuropathy” has been used to describe a wide group of disorders with symptoms of laryngeal irritation such as throat irritation, dysphonia, foreign body sensation in the throat, stridor and chronic cough.^1^, ^2^ But many, if not all of these symptoms, have also been found to be associated with LPRD. Are we, in many patients, really talking about a unifying underlying pathophysiologic process associated with dysfunction of the vagus nerve? Chronic vagal dysfunction of the upper respiratory and GI tracts, with distinct symptomology depending on which area of the vagus is most significantly affected. Rather than only “Chronic laryngopharyngeal neuropathy” is it not a broader entity, “Chronic upper respiratory and gastro-enterologic vagal/hypersensitivity neuropathy”?

Several causes have been identified as pharyngolaryngeal reflux, post nasal drip, allergy and asthma. In some cases the tests and treatments for these causes are performed and the patients persist with symptoms, especially chronic cough. And the suspicion of a sensory disorder in the laryngeal branches of the vagus nerve is a consideration. The etiology of a vagal neuropathy is uncertain, but commonly occurs after a viral infection.^3^ According to these authors, a neuropathic inflammatory process, such as Bell's palsy, trigeminal neuralgia and glossopharyngeal neuralgia, would occur in the vagus nerve.

Chronic laryngopharyngeal neuropathy (CLN) is a disease that may be present in otorhinolaryngologists’ offices, and is associated with laryngeal irritation such as throat irritation, dysphonia, foreign body sensation in the throat, stridor and especially chronic cough.^1^, ^2^ The vagus nerve presents an extensive innervation of the aerodigestive tract, including the upper and lower respiratory tract and aerodigestive tract. The neurological disorder can cause changes in the afferent branches of the reflex laryngeal and digestive reflex arch and various stimuli such as acid can trigger the symptoms.^2^, ^4^ Chronic laryngeal neuropathy may be associated with paradoxical vocal fold movement (PVFM) as part of an irritable laryngeal syndrome where afferent reflex hypersensitivity is a common mechanism.^5^, ^6^

CLN is often a diagnosis of exclusion. The patients may present with chronic cough, throat clearing, dysphonia or laryngospasm. The most common causes of these symptoms are infection, smoking, allergy, rhinosinusitis, bronchitis, asthma, cough associated with eosinophilic airway inflammation, gastro esophageal reflux disease (GER) and medication use, primarily ACE inhibitors. After exclusion of these causes, neuropathy should be considered.^1^

In the larynx, the cough reflex and laryngeal closure are important mechanisms of protection of the airways, the same receptors of the larynx are also found in the trachea and the larger airways and respond to the same pressure stimuli or irritants. Therefore, shortness of breath may be a common symptom in patients with predominantly laryngeal symptoms.^2^, ^7^ In patients with laryngeal neuropathy there is a sensitization of the cough reflex and a neuropathic response to the receptor stimuli. In the case, a stimulus such as an acute episode of allergy, a virus, a nonspecific irritant or a pharyngolaryngeal reflux (LPR) may develop a chronic cough due to a laryngeal sensory defect.^2^

The concept of irritable larynx was introduced by Morrison et al.^6^ Over the years other terms such as post-viral vagal neuropathy^3^, ^8^ and laryngeal sensory neuropathy^5^, ^9^, ^10^ have been used to the same suspected cause: vagal neuropathy. The etiology of neuropathy is uncertain, but viral infection is a cause suggested by several authors.^3^, ^11^ The result is a hypersensitivity of the cough reflex. It is not perfectly clear what begins chronic cough but often times it is noxious stimuli such as allergy, virus or reflux that begins the cycle^12^, ^13^ and although it may explain the origins it does not explain why cough then occurs not in the presence of such stimuli. It appears that there is change in central sensitization that is propagated via the vagus nerve, spinal cord and other descending pathways that allows for persistence of the over excitability.^14^, ^15^

A vagal neuropathy can also affect other motor branches of the vagus nerve, resulting in paresis or paralysis of the vocal folds, paradoxical vocal fold movement (PVFM), or other sensory branches inducing chronic cough, throat tickle, globus, sore throat, laryngeal paresthesia and laryngospasm. These symptoms may be aggravated by stimuli such as phonation, laughter, irritating inhalants, and laryngeal palpation.^2^

Although pharyngeal and laryngeal symptoms tend to be what brings these patients into the otolaryngologist's office, the vagus nerve also has extensive innervation of the aerodigestive tract, including the upper and lower respiratory tract and digestive tract. Vagal dysfunction can cause changes in the afferent branches of the reflex laryngeal and digestive reflex arch and various stimuli such as acid can trigger symptoms.^2^, ^4^

Most every aspect of normal swallowing, digestion and motility of the upper GI tract rely on normal vagal function. Not only does the vagus play a critical role in laryngeal, pharyngeal and laryngeal function during swallowing, the vagus is responsible for esophageal motility and peristalsis which allows proper movement of food down the esophagus into the stomach. The vagus nerve triggers acid secretion in the stomach for proper digestion of food and controls the pyloric valve to allow food to exit the stomach into the intestines The gall bladder stores bile which when released, assists in the proper digestion of food. Gall bladder function and the release of bile is under control of the vagus nerve (both directly and indirectly) and pancreatic secretion of enzymes that assist in digestion and absorption of nutrients, especially fats and proteins, is partially controlled by the parasympathetic fibers originating in the dorsal vagal nucleus and the nucleus ambiguous, then carried by the vagus nerve.^16^, ^17^ The vagus nerve stimulates the Sphincter of Oddi to open, allowing bile and pancreatic digestive enzymes to pass into the intestines. The vagus nerve also stimulates intestinal peristalsis and poor peristalsis can result in poor gastric emptying, gastroparesis, constipation, bloating and discomfort.^16^, ^17^ Vagal dysfunction can play an important role in both GERD and LPRD through reduced esophageal motility and delayed gastric emptying.

Although there are many causes for reflux there are a group of patients with typical heartburn symptoms but normal upper endoscopy and esophageal biopsies, normal esophageal pH test and a close correlation between patients’ heartburn and reflux events. Such patients are diagnosed with reflux hypersensitivity. “Reflux hypersensitivity is very common and together with functional heartburn accounts for more than 90% of the heartburn patients who failed treatment with proton pump inhibitor twice daily”.^18^ Although surgery and reflux therapy may play a role in these patients, reflux hypersensitivity is primarily treated with esophageal neuromodulators, such as tricyclic anti-depressants and selective serotonin reuptake inhibitors among others.^18^ Sensory neuromodulators are also one of the potential treatments of gastroparesis.^19^

This leads back to the question, are there patients with varied symptoms that have a common physiologic abnormality either directly through the vagus (vagal neuropathy) or in the central nervous system (central sensitization) that results in aberrant information being sent via descending pathway circuits through the vagus and spinal cord? Clearly, there are patients who have specific causes for their dysfunction and do not have a generalizable vagal neuropathy or central/peripheral hypersensitivity. There are patients with primary LPRD, allergic airway disease, rhinosinusitis and asthma, autoimmune disorders, environmental or microbiological irritation/inflammation or habitual behaviors that induce inflammation. These patients are often relatively easy to tease out and treat. It is the chronic patient who has been assessed and treated for a number of possible disorders such as LPRD, asthma and allergy but who have not responded that may have a more generalizable hypersensitivity reaction. It is in these patients where it may be prudent to do an early trial of a neuromodulator such as gabapentin, which has both central and descending pathway action or the tricyclic antidepressants which are both serotonin-norepinephrine reuptake inhibitors and also work on the descending pathways.^20^ These neuromodulators are believed to treat the hypersensitivity and central sensitization observed in patients with multiple symptoms including chronic cough and laryngeal hypersensitivity, as they do for patients with neuropathic pain, and they are becoming a bigger part of the practice of laryngologists and otolaryngologists. Gabapentin is currently recommended by the American College of Chest Physicians for use in unexplained chronic cough while the tricyclic antidepressants have been found to be effective in both chronic cough^21^ and in globus where amitriptyline was found to be more effective than reflux treatemnt.^22^

The bottom line is that there are some common neurologic themes in many of these somewhat disparate head and neck complaints and that there may be an underlying unifying process: “Chronic laryngopharyngeal hypersensitivity or vagal neuropathy”. Although treating the underlying specific conditions and symptoms are needed, failure of therapy should lead to a consideration of treatment with a neuromodulator, and this may not only lead to a more definitive diagnosis it may provide relief to the patients that they have often sought for a long time.

## Conflicts of interest

The authors declare no conflicts of interest.
